# Biochemical and structural characterization of tyrosine aminotransferase suggests broad substrate specificity and a two‐state folding mechanism in *Leishmania donovani*

**DOI:** 10.1002/2211-5463.12715

**Published:** 2019-08-31

**Authors:** Santanu Sasidharan, Prakash Saudagar

**Affiliations:** ^1^ Department of Biotechnology National Institute of Technology Warangal India

**Keywords:** folding studies, *Leishmania donovani*, molecular dynamic simulation, pyridoxal‐5‐phosphate, Tyrosine aminotransferase

## Abstract

Tyrosine aminotransferase (TAT) is an aminotransferase with broad substrate specificity that catalyzes the transamination of aromatic amino acids in *Leishmania donovani* and plays a crucial role in the survival and pathogenicity of the parasite. In this study, we have biochemically characterized tyrosine aminotransferase from *Leishmania donovani* using *in vitro* and *in silico* techniques. *Leishmania donovani* tyrosine aminotransferase (LdTAT) was cloned into the pET28a(+) vector and expressed in the BL21 strain of *Escherichia coli*. The Ni‐NTA‐purified protein was then characterized biochemically, and its various kinetic parameters were investigated. The apparent *K*_m_ value for the tyrosine–pyruvate pair was determined to be 3.5 ± 0.9 mm, and *V*_max_ was analyzed to be at 11.7 ± 1.5 μm·min.μg^−1^. LdTAT was found to exhibit maximum activity at 50 °C and at a pH of 8.0. Cofactor identification for LdTAT showed that pyridoxal‐5‐phosphate (PLP) binds with a *K*_m_ value of 23.59 ± 3.99 μm and that the phosphate group is vital for the activity of the enzyme. Sequence analysis revealed that S151, Y256, K286, and P291 are conserved residues and form hydrogen bonds with PLP. Urea‐based denaturation studies revealed a biphasic folding mechanism involving N→X→D states. Molecular dynamic simulations of modeled LdTAT at various conditions were performed to understand enzyme behavior and interactions at the molecular level. The biochemical and structural divergence between host and parasite TAT suggests the LdTAT has evolved to utilize pyruvate rather than α‐ketoglutarate as co‐substrate. Furthermore, our data suggest that LdTAT may be a potential drug target due to its divergence in structure and substrate specificity from the host.

**Abbreviations**
KMB: α‐ketomethiobutyrate
KMV: 2‐keto‐3‐methyl‐valerate
LdTAT: *Leishmania donovani* tyrosine aminotransferase
MD: molecular dynamics
NADH: nicotinamide adenine dinucleotide
PLP: pyridoxal‐l‐phosphate
Rg: radius of gyration
RMSD: root mean square deviation
RMSF: root mean square fluctuation
SASA: solvent accessible surface area
TAT: tyrosine aminotransferase

Leishmaniasis is a group of diseases caused by protozoan parasites that span over 20 *Leishmania* species. According to WHO (2018), 0.7–1 million cases are reported world over every year with 20 000–30 000 deaths occurring annually. The disease prevails mainly in Brazil, Africa, Afghanistan, Algeria, Columbia, Iran, and the Indian subcontinent. The parasite manifests itself in three forms, namely visceral leishmaniasis, cutaneous leishmaniasis, and mucocutaneous leishmaniasis 1. *Leishmania donovani* (Kinetoplastida: Trypanosomatidae), also known as kala‐azar, is the major causative agent of visceral leishmaniasis, which is fatal when left untreated. *Leishmania* possesses a dimorphic life cycle that alternates between extracellular motile promastigotes and immobile intracellular amastigotes 2. Transmission of the parasite takes place during the bite of an insect vector belonging to subfamily: Phlebotominae. On infecting a human host, the parasite invades the macrophages and transforms into nonmotile and afflagelated amastigotes. This complex life cycle of *Leishmania* parasite makes it difficult to prevent transmission or infection of the disease.

Leishmaniasis is currently treated by the injection of antimony compounds and miltefosine 3. Another antimicrobial drug amphotericin B is also used as an alternative treatment option in India, especially its liposomal formulation 4. The relapse of infection after treatment with pentavalent antimonial compounds, reduction in efficacy of miltefosine, renal toxicity of amphotericin B, and rising drug resistance predispose the requirement for new drug compounds and above all, new drug targets 5. The need for well‐characterized drug targets drove us to the study on tyrosine aminotransferase.

During the metacyclogenesis of the parasite, promastigotes from the amino acid‐rich gut of insect differentiate into amastigotes occupying nutrient‐limited phagolysosomes 6. To compensate for the dearth of energy and nutrients, catabolism of amino acids is initiated for NADH re‐oxidation and methionine recycling. This crucial pathway of amino acid catabolism in trypanosomatids takes place in two steps where the initial step involves the reversible transamination of aromatic amino acid to its respective oxo‐acid. The amino group of the aromatic amino acid is transferred to the approaching oxo‐acid and converted to its respective amino acid. The deaminated amino acid (L‐2‐hydroxy acid) is reduced by dehydrogenase enzyme and excreted eventually. The primary step of this pathway is catalyzed by a broad specificity tyrosine aminotransferase in the parasite 7.

Tyrosine aminotransferase (L‐tyrosine: 2 oxoglutarate aminotransferase; EC 2.6.1.5; TATase) is a homodimer that belongs to the fold type I aminotransferases and lies in the PLP (Pyridoxal‐l‐phosphate)‐dependent superfamily. The enzyme has been characterized earlier in other organisms and has been identified in playing a vital role in many pathways. The role of this enzyme is attributed to pathogenesis in other trypanosomatids, especially in *Trypanosoma brucei* 8. The depletion of amino acids in the parasite can result in the deficiency of essential metabolites in the host. Stibbs and Seed also related the pathogeny of the *Trypanosoma brucei* to the phenylpyruvate end products 9. The detection of high levels of aromatic amino acid oxidation end products in the supernatant of *Trypanosoma cruzi* epimastigotes was also linked to pathogenicity, and its role in re‐oxidation of NADH was also elucidated 10. *Trypanosoma cruzi* tyrosine aminotransferase is responsible for the conversion of pyruvate to alanine that is secreted out of the parasite. Other transamination products like 4‐hydroxyphenylpyruvate are reduced subsequently to aromatic lactates by dehydrogenases. This reaction leads to the re‐oxidation of cytosolic NADH 10, 11, 12. These routes are found to compensate for the lowered activity in Krebs cycle and respiratory chain 13. On the other hand, mammalian tyrosine aminotransferase maintains the tyrosine concentration at subtoxic levels with the help of α‐ketoglutarate.

The methionine‐recycling pathway is yet another route that is catalyzed by this broadly specific aminotransferase in *Trypanosoma brucei* and *Crithidia fasciculata*. The enzyme in the pathway catalyzes the last step of converting α‐ketomethiobutyrate (KMB) to methionine via transamination process 14. Methionine is an essential amino acid in fast‐growing cells, and therefore, this pathway has been targeted before as antimalarial and anti‐*T. brucei* in various therapies 15, 16. Tyrosine aminotransferase is also capable of transaminating 2‐keto‐3‐methyl‐valerate (KMV) to give valine as the end product. It has been found that valine is an essential amino acid that is required for the survival of the parasite in the dimorphic cycle 17. These data suggest that the LdTAT may also play an important role in the infectivity of the parasite while providing resistance against contemporary drugs.

LdTAT catalyzes the transamination of numerous amino acids, and therefore, it holds huge potential as a drug target. To utilize an enzyme as a drug target, the complete biochemical characterization holds the key to design better drugs. In this study, tyrosine aminotransferase from *Leishmania donovani* was cloned, expressed, and purified. The biochemical characterizations such as kinetic studies, cofactor identification, the study of the effect of pH, and temperature were also carried out. In addition to this, urea denaturation studies were also undertaken to understand the enzyme's reactivity in the presence of a chaotropic agent and its folding mechanism. To corroborate the above findings, modeling and molecular dynamics simulation studies were also carried out to identify the molecular interactions underlying the mechanisms. These biochemical studies backed by *in silico* analysis aim to validate this enzyme as a potential candidate for drug target studies against the parasite while keeping the host unharmed.

## Results

### Sequence analysis

**Figure 1:**
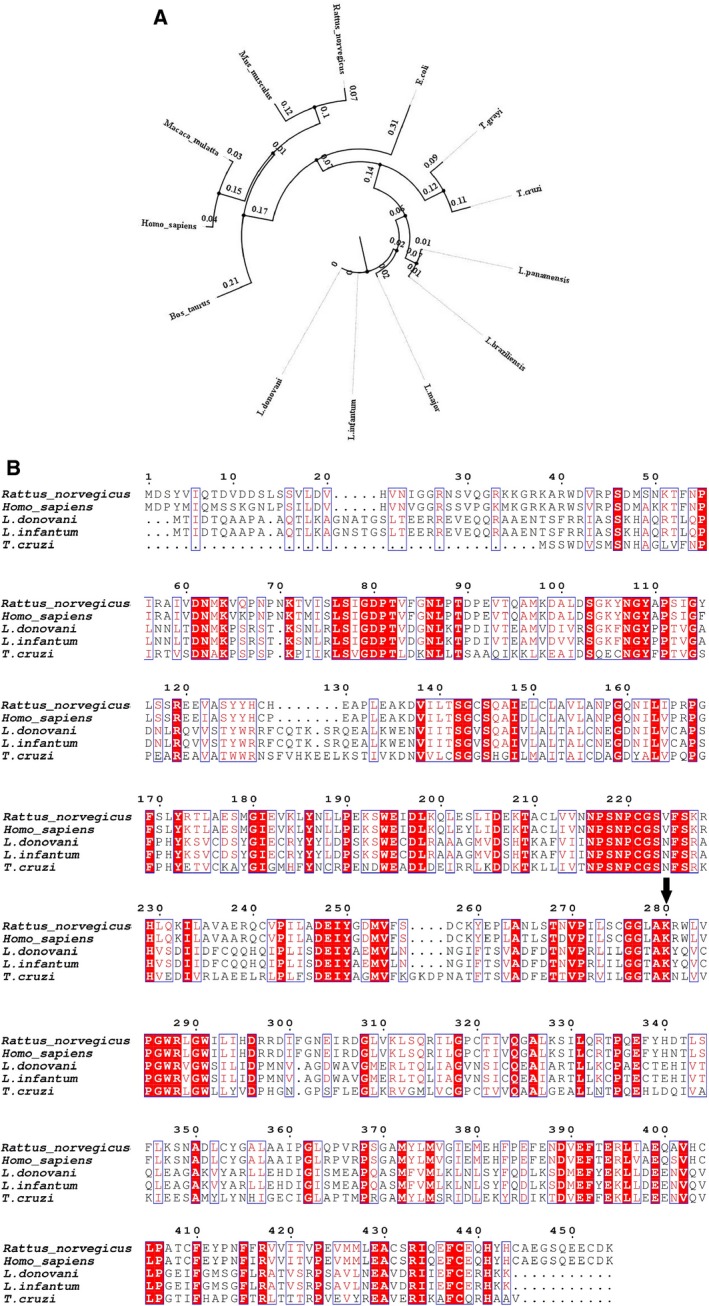
Sequence analysis of LdTAT (A) Clustal Omega analysis represented in the form of a tree showing genetic relatedness of LdTAT gene sequence with tyrosine aminotransferases of other organisms. The tree shows the distant relatedness of the LdTAT genetic sequence to human tyrosine aminotransferase. (B) Multiple sequence alignment of tyrosine aminotransferase sequences of LdTAT and other related organisms. Red highlighted boxes represent identical and conserved amino acid residues, and nonhighlighted boxes represent similar amino acid residues. Arrow depicts the active site residue K286 that is conserved across all the sequences.

The gene is located on chromosome 36 and is present as diploid. Phylogeny analysis (Fig. 1A) found that LdTAT gene sequence is closely related to *Leishmania infantum*. TAT from Trypanosoma was found to be next closely related genus followed by *Escherichia coli*. Mammalian TAT is distantly related to LdTAT and other parasitic TAT. At the protein sequence level, multiple alignment of sequences showed conserved regions at various sites (Fig. 1B). Protein sequence of LdTAT is only 35% similar to rat and human TAT, whereas *Trypanosoma cruzi* shares 40% identity with LdTAT. K286 was found to be conserved among the protein sequences, which plays a vital role as the anchoring residue for the cofactor. N‐terminal region showed very few conserved residues, while C‐terminal displayed several charged residues like D389, E391, E394, R417, and E429, and conserved throughout. The decreased sequence identity between organisms could be a reason that makes LdTAT unique in its choice of excluding α‐ketoglutarate as a co‐substrate. Three‐dimensional structural comparison of LdTAT and human TAT (PDB ID: 3DYD) showed that the N‐terminal region varied widely from the human TAT. It was also found that LdTAT exhibits higher α‐helix content when compared to human TAT. The solvent‐accessible area for LdTAT was higher (22035.70 Å^2^) when compared to human TAT (18012.22 Å^2^). Apart from the binding site, LdTAT houses cavities near the N‐terminal (P80‐R126) and C‐terminal region (L350‐G410) of the protein, which is devoid in the human TAT.

### Cloning, expression, and purification of LdTAT

**Figure 2:**
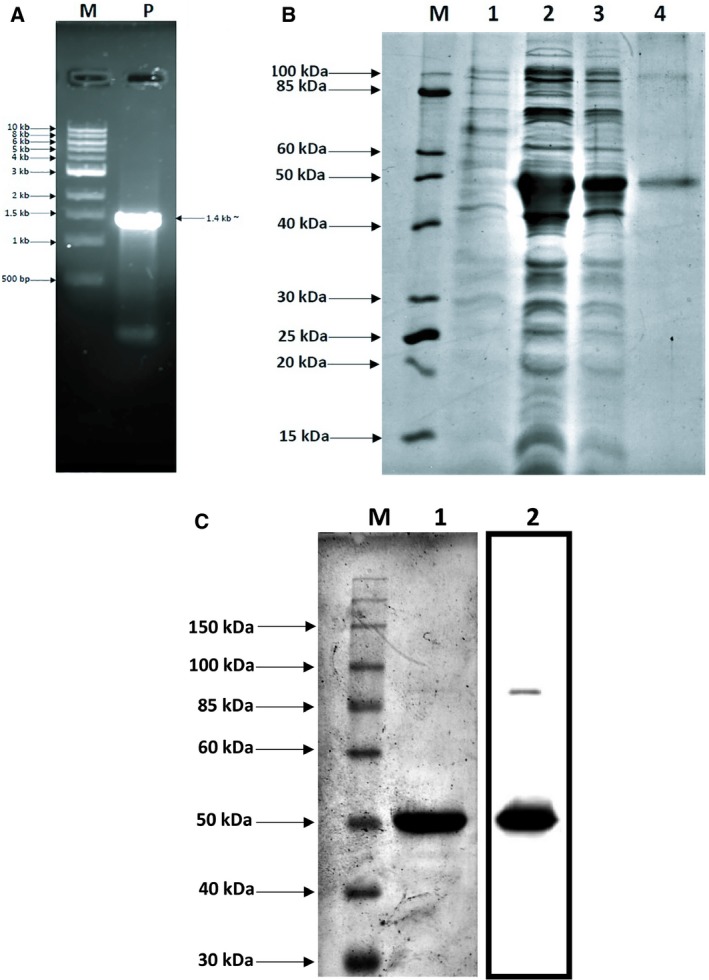
Cloning and expression of LdTAT (A) Gene amplification of LdTAT sequence (1347 bp) Lane M: 10 kb marker (NEB). Lane P: PCR product at 1347 bp. (B) Protein expression on 12% SDS/PAGE. Lane M: 10–200 kDa protein ladder (NEB). Lane 1: Uninduced LdTAT BL21 cells. Lane 2: Sonicated supernatant showing LdTAT BL21 induction. Lane 3: Flowthrough after Ni‐NTA binding. Lane 4: Purified protein showing expression at 49.3 kD and a dimer was observed at 98.6 kDa. (C) Western blot with anti‐His antibody. Lane 1: 10–200 kDa protein ladder. Lane 2: 12% SDS/PAGE gel with eluted protein. Lane 3: Anti‐His blots showing luminescent bands at 48 and 96 kDa. Uncropped gel and blot images are represented in Fig. S2.

The PCR amplification of LdTAT yielded a band at 1347 bp (Fig. 2A). The cloning of LdTAT was confirmed by the amplification through PCR and restriction digestion of pET28a(+) construct using *Bam*HI and *Hind*III. Sequence and frame of the insert in the construct was confirmed by sequencing. The sequence was deposited to GenBank, and GenBank id: MK426678 was assigned**.** Protein expression in *E. coli* BL21 was successfully optimized, and molecular weight of expressed LdTAT was confirmed by running the protein on a 12% SDS/PAGE gel (Fig. 2B). The molecular mass of the protein was found to be 49.3 kDa, and another band around 98.6 kDa was also observed. The molecular weight of the second band corresponded to the homodimer of the LdTAT. Western blot analysis using anti‐His antibody also showed two bands around the molecular weight of 49.3 and 98.6 kDa, thus confirming the expression and purification of LdTAT (Fig. 2C).

### Modeling, docking, and dynamic Simulation of LdTAT

**Figure 3:**
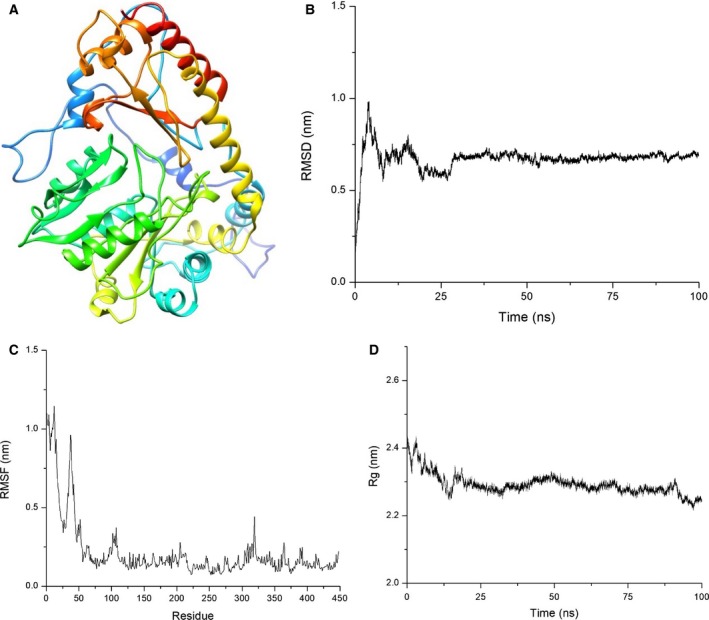
Modeling and Energy Minimization (A) Energy minimized structure of LdTAT. (B) RMSD analysis of Cα atoms exhibited an average value of 0.67 ± 6e‐4 nm for 100‐ns production MD showing stable structure. (C) RMSF analysis of the residues showed N‐terminal fluctuation and fluctuation in the 280–325 residue housing the active site. (D) Rg calculation of Cα atoms, with an average of 2.29 ± 3e‐4 nm, also corroborated the structural rigidity and folding compactness of the modeled protein LdTAT.

The modeled LdTAT PM0081305 was analyzed for structural integrity using RAMPAGE and SAVES servers. Ramachandran plot analysis for the model showed 98.2% of the residues in the favored region, 1.3% in the allowed region, and 0.4% in the outlier region. PROSA score of PM0081305 was −8.56, and the score remained in the region of known native structures. The template structure that was taken for modeling (PDB ID: 4IX8) had a PROSA score of −8.89. The structure was then minimized using Amber99 force field and conjugate gradient method to a minimum of −1.89 × e6 kJ·mol^−1^ (Fig. 3A). The production MD was then performed for 100 ns to determine the integrity and stability of modeled structure. RMSD value for the Cα atoms of PM0081305 was calculated to be 0.67 ± 6e‐4 nm, while RMSF calculations had fluctuations in the N‐terminal and around conserved domains of the protein. Rg measurements gave a value of 2.29 ± 3e‐4 nm that remained stable throughout. The values showed that the modeled structure was stable and folded properly and therefore can be utilized for further *in silico* studies (Fig. 3B–D).

### Kinetic characterization of LdTAT

**Figure 4:**
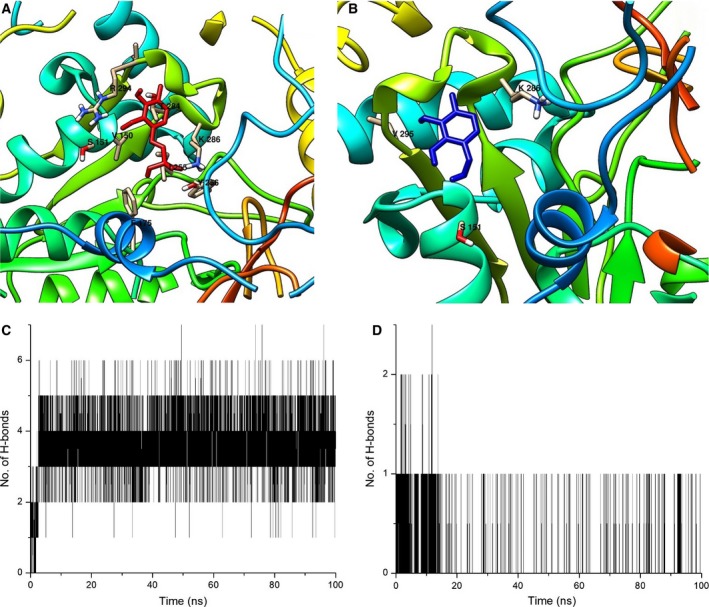
PLP and Pyridoxal cofactor identification (A) PLP (in red) binding in the active site cavity with residue K286, which is the anchoring residue just beside the PLP cofactor. Other amino acids S151 and Y256 are also found to be the vicinity. (B) Pyridoxal (In blue) showed binding with only S151 and V295. Pyridoxal was found to be away from the K286 residues and other binding residues. (C) Production MD of 100 ns showing hydrogen bond formation between LdTAT and PLP. The number of hydrogen bonds was found to be between 2 and 6 on an average. (D) Production MD of 100 ns showing poor hydrogen bond formation between pyridoxal and LdTAT protein.

Kinetic studies on LdTAT showed that the enzyme is a broad aminotransferase capable of utilizing tyrosine, phenylalanine, and tryptophan as substrates. Tyrosine as substrate displayed a *K*_m_ value of 3.5 ± 0.9 mm and *V*_max_ of 11.7 ± 1.51 μm·min.μg^−1^. The study also found that phenylalanine and tryptophan have a *K*_m_ of 3.752 ± 0.96 mm and 5.055 ± 0.42 mm, respectively. Among the keto‐acids that were tested, KMB with tyrosine showed a very high binding affinity toward the enzyme with a *K*_m_ value of 0.8103 ± 0.11 μm, whereas sodium pyruvate with tyrosine was found to have 42.67 ± 0.38 mm *K*_m_ value. α‐ketoglutarate failed to show any activity against any of the aromatic amino acids (Table 1). The Michaelis‐Menten plot for various substrate–co‐substrate pair is shown in Fig. S1. We also studied LdTAT's compliance with PLP and pyridoxal compounds as cofactor molecules. PLP displayed affinity toward LdTAT with a *K*_m_ value of 23.59 ± 3.99 μm and *V*_max_ of 7.816 ± 0.35 μm·min.μg^−1^, whereas pyridoxal failed to catalyze the reaction. Docking and simulation studies over 100 ns displayed similar fates for PLP and pyridoxal. On docking, PLP had higher binding to LdTAT with a binding energy of −6.26 kcal·mol^−1^ compared to −5.33 kcal·mol^−1^ of pyridoxal. PLP was able to form hydrogen bonds with S151, Y256, K286, and P291 (Fig. 4A), wherein pyridoxal was found to bind to S151 and V295 (Fig. 4B). Hydrogen bond analysis over 100‐ns production MD showed 4–6 hydrogen bonds between LdTAT and PLP, whereas pyridoxal formed poor and intermittent hydrogen bonds of an average 1 with LdTAT (Fig. 4C,D).

**Table 1:** Substrate–co‐substrate utilized and apparent *K*_m_ and *V*_max_ values determined for the various pairs. Tyrosine exhibited the highest activity among the substrates, and KMB co‐substrate displayed higher affinity toward LdTAT. KG failed to show any activity in the assay. SP—Sodium pyruvate; Tyr—Tyrosine; KG—α‐ketoglutarate; KMB—α‐ketomethiobutyrate; Phe—Phenylalanine; Trp—Tryptophan

Substrate	Co‐substrate	*K*_m_	*V*_max_ (μm·min.μg^−1^)
SP (100 mm)	Tyr (0–7) mm	3.5 ± 0.90 mm	11.7 ± 1.51
Tyr (4 mm)	SP (0–200) mm	42.67 ± 0.38 mm	8.6 ± 5.8
KG (125 mm)	Tyr (0–7) mm	–	–
Tyr (4 mm)	KG (0–200) mm	–	–
KMB (1.25 μm)	Tyr (0–7) mm	0.7809 ± 0.06 mm	1.462 ± 0.03
Tyr (4 mm)	KMB (0–4.5 μm)	0.8103 ± 0.11 μm	1.854 ± 0.07
SP (100 mm)	Phe (0–7) mm	3.752 ± 0.96 mm	19.79 ± 2.60
SP (100 mm)	Trp (0–7) mm	5.055 ± 0.42 mm	19.94 ± 0.94

### Effect of temperature and pH on LdTAT

**Figure 5:**
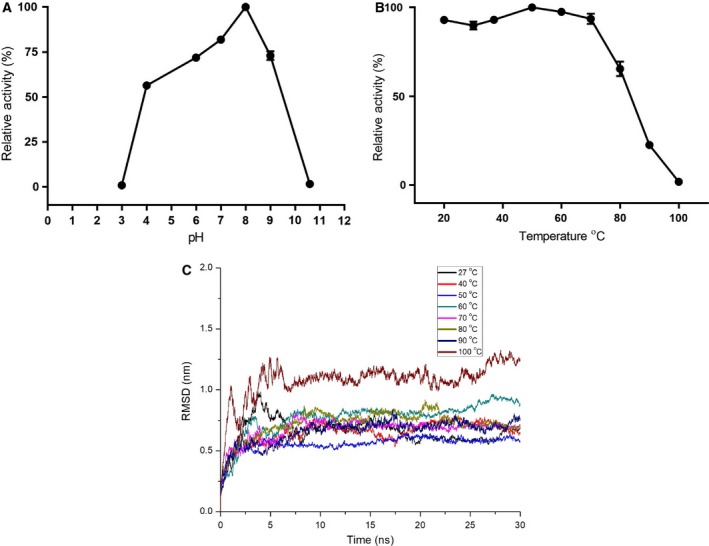
pH and temperature characterization. (A) Activity analysis at different pH found that the enzyme is stable at 8 with a decrease in activity toward both alkaline and acidic pH. (B) The enzyme activity was found to be stable till 50 °C after which the activity declined. (C) Production MD showed that the structure remained stable till 50–60 °C, after which there were huge deviations in the RMSD of the structure as shown in the figure. Error bars represent standard deviation.

Relative activity of LdTAT was calculated for different temperature and pH. LdTAT was found to have the highest activity at a pH of 8. Toward the acidic and basic pH, LdTAT showed a gradual loss of activity (Fig. 5A). In the pH range of 6–9, the activity loss was found to be only 20–30%. Studies on temperature stability revealed that LdTAT showed greater temperature stability. The highest activity was recorded at a temperature of 50 °C, and an average 10% loss of activity was recorded between 20 and 50 °C (Fig. 5B). Complete loss of activity was registered only when the assay was performed at 100 °C. To understand more on the temperature stability, a 30‐ns production MD was run at different temperatures. RMSD value for Cα atoms of LdTAT showed the best stability at 50 °C with a value of 0.559 ± 0.0011 nm. Interestingly, the calculated RMSD tended to increase after 50 °C with the average value differing by > 0.2 nm. Production MD of modeled LdTAT at 100 °C had RMSD values that shot > 1 nm (1.1 ± 0.002 nm). The behavior of modeled LdTAT at different temperature substantiated the activity analysis, and the increased temperature might have caused structure denaturation (Fig. 5C).

### Urea denaturation studies on LdTAT

**Figure 6:**
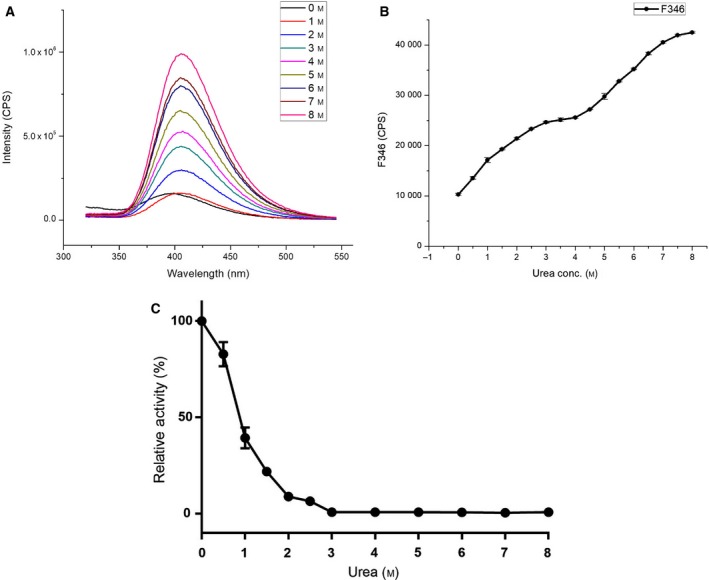
Urea denaturation studies (A) Fluorescence intensity measured at various urea concentration. A steep increase and redshift were observed in the graphical representation. (B) The increase in the fluorescence intensity at 346 nm showing N→X→D biphasic based folding mechanism. (C) Relative activity of LdTAT with increasing urea concentration was found to decrease till 3 m urea concentration. Complete loss of activity was found at a urea concentration of 4 m that corresponds to the unfolding mechanism in (B).

**Figure 7:**
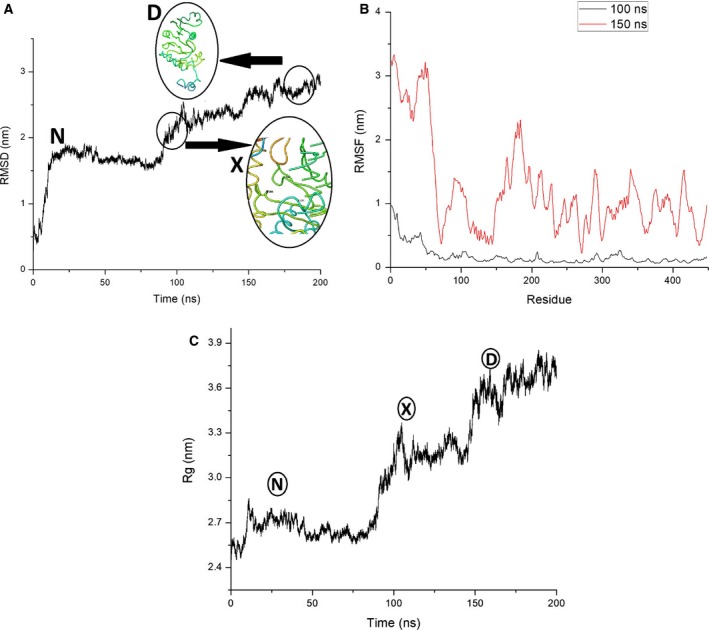
Folding mechanism of LdTAT. (A) RMSD analysis of LdTAT over 200 ns showed the three‐state unfolding mechanism of LdTAT N→X→D. The trajectory analysis showed that the active site is intact at X state at 100 ns. At 150 ns, the active site is completely lost and the secondary structure of LdTAT is completely lost. Active site structure is shown in the inset circle. (B) RMSF analysis found that the N‐terminal region fluctuation is high and the region between 150 and 200 amino acid residues. At 150 ns, the complete structure possesses huge fluctuation depicting the structural loss. (C) Rg analysis, which determined the protein fold compactness, verified the three‐state biphasic folding mechanism. The N stage, X stage, and D stage are depicted in black circles.

Urea denaturation studies involved the calculation of relative activity and fluorescence intensity at various urea concentration. LdTAT showed a gradual decrease in the activity till 2.5 m urea, after which the complete loss of activity was measured (Fig. 6C). Tyr, Trp, and Phe amino acids are present either partially or fully buried in the hydrophobic core of the folded proteins, or they are found in the interface of multidomain proteins. Fluorescence of the Trp residues showed an increase in the fluorescence intensity till 4 m urea and then a gradual increase to saturation at 6 m urea. A redshift was also observed with an increase in urea concentration (Fig. 6A). The fluorescence intensity analysis at 346 nm showed an intermediate state that forms between 3 and 4 m urea before denaturing completely at 6 m urea (Fig. 6B). Therefore, we hypothesized a biphasic system of unfolding, wherein N→X→D: N, X, and D are native, intermediate, and denatured states, respectively. To understand more on the unfolding pathway at the molecular level, a production MD at 8 m urea was run for 200 ns and the RMSD analysis showed a sudden intermediate incline at 100 ns and 150 ns (Fig. 7A). RMSD value for the N state showed 1.58 ± 0.003 nm, whereas X and D states showed average RMSD value > 0.6 nm from N state. The trajectory analysis at 150 ns showed the complete rupture of the active site pocket, and hence, the loss of activity was observed. The RMSF analysis at 150 ns showed increased fluctuation (> 2 nm) when compared to 100‐ns RMSF (Fig. 7B). This was due to the completed loss of structural integrity at the residue level at 150 ns. Rg value was averaged to be 2.65 ± 7e‐4, 3.06 ± 0.002 nm, and 3.6 ± 0.0017 nm for N, X, and D states, respectively. The change in the Rg value of > 0.5 nm also verified the presence of an intermediate X state. Furthermore, the SASA measured for these three phases and found to be 214 ± 0.12 nm·S^−2^. N, 274 ± 0.12 nm·S^−2^. N, and 304 ± 0.12 nm·S^−2^. N, respectively (Fig. 7C). SASA analysis showing increased average calculated value supported the folding mechanism in three stages, and the increase is due to the accessibility of solvent upon denaturation (Fig. S2). Therefore, the amalgamation of the measured results allowed us to hypothesize the biphasic nature of folding mechanism in LdTAT consisting of three states.

## Discussion

**Figure 8:**
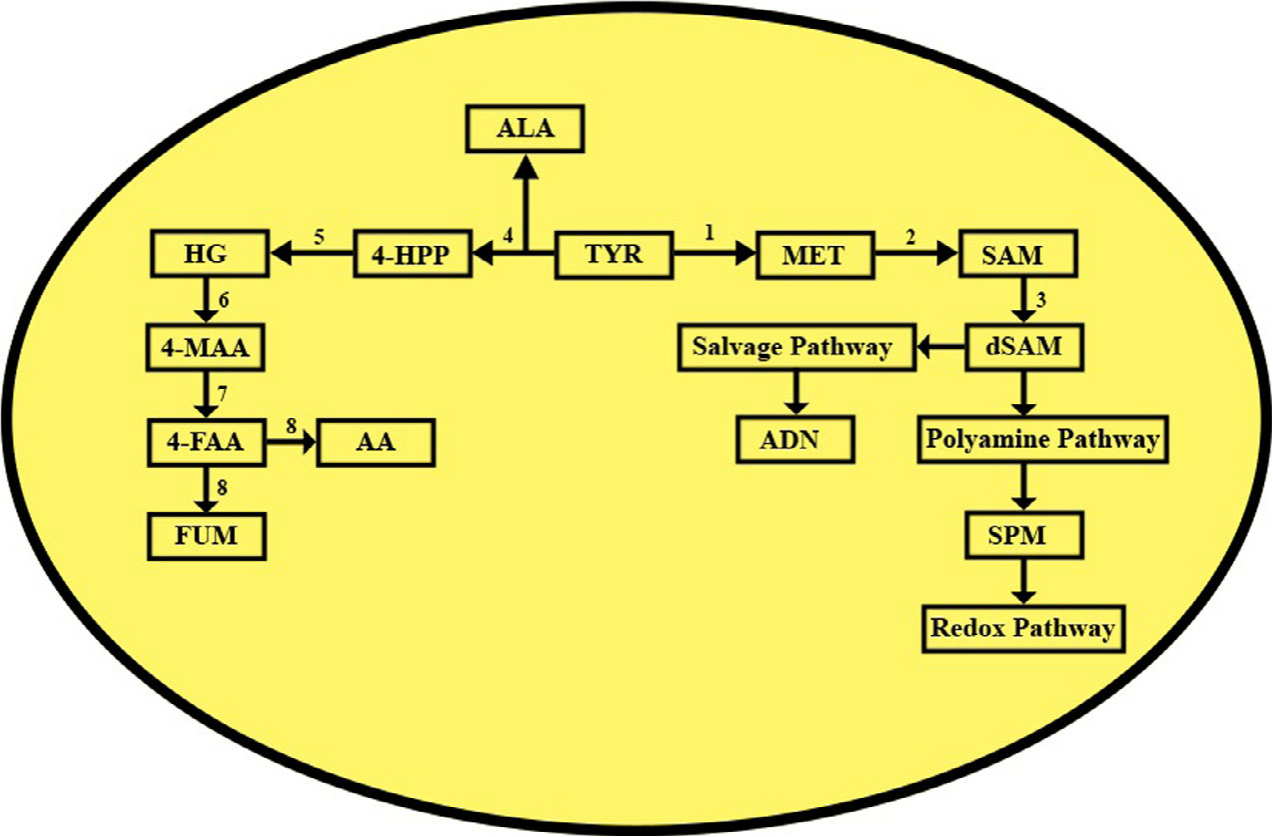
Different fates of Tyrosine aminotransferase pathway: Figure showed the fates of different substrates and co‐substrates that are utilized by LdTAT and the other vital pathways that are related. 4‐FAA, 4‐Fumarylacetoacetate; 4‐HPP, 4‐Hydroxy phenylpyruvate; 4‐MAA, 4‐maleylacetoacetate; AA, Acetoacetate; ADN, Adenine; dSAM, decarboxylated S‐adenosyl methionine; FUM, Fumarate; HG, Homogentisate; MET, Methionine; SAM, S‐adenosyl methionine; SPM, Spermine; TYR, Tyrosine. 1/4‐Tyrosine aminotransferase; 2‐SAM synthetase; 3‐SAM decarboxylase; 5‐HPP dehydrogenase; 6‐Homogentisate dioxygenase; 7‐Maleylacetoacetate *cis‐trans‐*isomerase; 8‐Fumarylacetoacetic acid hydrolase.

Tyrosine aminotransferase is a well‐characterized enzyme in many organisms, but the characterization of the enzyme in *Leishmania donovani* has not been ventured yet. LdTAT also plays a major role in the metabolism inside the parasite and is interconnected to various important pathways (Fig. 8). Polyamine and salvage pathways are principal routes that maintain the nucleotide and redox concentrations in the parasite. Moreover, fumarate and acetoacetic acid also play vital roles in glucose metabolism and the Krebs cycle. The requirement of any drug target is its complete characterization at all levels. Tyrosine aminotransferase plays many important roles in metabolic pathways inside the *Leishmania* parasite, and therefore, this study was conducted to unravel the biochemical characteristics of tyrosine aminotransferase.

From the sequence analysis, we found that LdTAT shares only 40% similarity with the human host. We also observed that within the *Leishmania* genus, few variations exist at the gene sequence level. When the protein sequence was analyzed in detail, K286 was found to be conserved through all the other tyrosine aminotransferases. In the N‐terminal domain, we found very few residues that shared identity but the C‐terminal had more conserved residues when compared. The amino acids that formed hydrogen bonds with PLP molecules such as S151, Y256, K286, and P291 were found to be conserved across all the species that were compared. Few amino acids around these conserved residues were also identical or similar, thus providing the structural ambiance required to house and electrostatically stabilize the PLP molecule. This uniqueness of LdTAT at sequence level from its host human tyrosine aminotransferase made it primarily qualify as a potential drug target. On cloning and expressing the 49.3 kDa LdTAT, the enzyme was found to exist both in its monomeric and dimeric form, and this was confirmed by western blotting using anti‐His antibody. Tyrosine aminotransferase has been known to exist in the dimeric form in other organisms too. PLP, being its natural cofactor, binds to the active site at the interface of the dimer protein.

For any enzyme, kinetic studies are essential to determine the substrate and co‐substrate specificities that are a direct measure of the pathways involved. LdTAT had exhibited specificities toward tyrosine, phenylalanine, and tryptophan with a higher affinity toward tyrosine. In humans, TAT exhibits affinity toward α‐ketoglutarate as co‐substrate. The Km was recorded to be 870 ± 160 mm when tyrosine was used as substrate and 3300 ± 600 mm when α‐ketoglutarate was the substrate alternatively. Phenylalanine failed to show any activity toward human TAT with α‐ketoglutarate 18. Among the co‐substrate that were employed, KMB displayed an increased affinity toward LdTAT leading to the synthesis of methionine. Methionine production plays a vital role in two pathways, the salvage and the polyamine. Salvage pathway leads to the production of adenine, a key nucleobase required for DNA replication. Polyamine pathway is essential for maintaining the free radical's equilibrium and ROS activity. The enzyme did not exhibit any activity to α‐ketoglutarate. This inactiveness to α‐ketoglutarate deviates LdTAT from the human tyrosine aminotransferase (HTAT). HTAT is widely known to be a broad aminotransferase, and α‐ketoglutarate is utilized by HTAT to give rise to glutamate. This absence of activity toward α‐ketoglutarate substantiated the druggable nature of LdTAT. Sobrado *et al*. by site‐directed mutagenesis found that R57A and R57Q mutations in rat tyrosine aminotransferase abolished its activity completely. Mutation in analogous residues of *T. cruzi* TAT*,* N17S, and R20A/Q led to lowered catalytic efficiency of mutated variant significantly 19. Therefore, the authors hypothesize that the N‐terminal region of LdTAT that has few identical residues may hold the reason for co‐substrate specificity. The study on the acceptance of pyridoxal as a cofactor was analyzed to understand the necessity of the phosphate tail group of PLP. PLP, even though a heavier molecule compared to pyridoxal, sits comfortably in the active site and is stabilized by the K286 anchor residue. Pyridoxal does not achieve this stabilization and therefore fails to initiate enzyme functionality. Pyridoxal‐based assay supplemented with 30 μm PLP regained LdTAT activity immediately. The MD simulation studies also substantiated the higher binding of PLP when compared to pyridoxal molecule. The study shows that the phosphate tail of PLP is essential for the enzyme activity and that enzyme is capable of housing large molecules that are structurally similar to the PLP.

The stability of the enzyme at various pH and temperature was carried out, and it was found that the enzyme is stable between 6 and 9 pH with the maximum activity at pH 8. The loss of activity at acidic pH might be due to nonbinding of PLP in the presence of excess H^+^ ions concentration, and at alkaline pH of 10, the enzyme losses secondary structure, thereby functionality. The temperature studies showed that the LdTAT has higher temperature stability and this has been reported in other tyrosine aminotransferases 20. LdTAT remained active till 50 °C, and RMSD analysis supported the study. The authors assume that the increased temperature stability might be due to the higher intramolecular H‐bonding in LdTAT and also due to the structural stability conferred by the binding of PLP cofactor. The structure–function co‐relationship of LdTAT is essential to study the effect of drugs at the binding site. Urea denaturation study of LdTAT cast light into the biphasic folding nature of LdTAT. The intermediate state of LdTAT was found to be the point of activity loss until the activity was found to decrease gradually. The authors theorize that the N‐terminal is required for the activity of LdTAT. The mechanism might be that the co‐substrate binds to the N‐terminal residues, which then swings toward the active site pocket at K286 for transamination. The high RMSF value at N‐terminal substantiates this claim although further in‐depth studies are required to support the mechanism. The mutation of N‐terminal residues has previously been known to cause loss of activity 19. Therefore, the unique N‐terminal might be the co‐substrate binding domain in LdTAT according to the folding studies.

This study biochemically characterized LdTAT both at *in vitro* and *in silico* levels. The low sequence similarity with host tyrosine aminotransferases, nonacceptance of host co‐substrate, and its increased affinity to methionine make it unique compared to other tyrosine aminotransferases. Furthermore, the necessity of phosphate tail of PLP throws light into the possible mechanism of transaminase activity. Temperature and pH studies also concluded the higher stability of LdTAT at physiological conditions. The biphasic folding mechanism of LdTAT has also widened our understanding of the transaminase mechanism of LdTAT that differs widely among organisms. In a nutshell, LdTAT holds immense potential to be carried forward as a drug target owing to its unique biochemical properties.

## Materials and methods

### Sequence analysis

The sequence of *Leishmania donovani* tyrosine aminotransferase (GenBank ID: 13386702) was obtained from the GenBank database 21 and aligned against tyrosine aminotransferase gene sequences of *Rattus norvegicus*,* Mus musculus*,* Macaca mulatta*,* Homo sapiens*,* Bos taurus*,* Escherichia coli, Trypanosoma grayi, Trypanosoma cruzi, Leishmania panamensis*,* Leishmania braziliensis*,* Leishmania infantum,* and *Leishmania major* by using clustal omega 22. A phylogenetic tree summarizing the genetic relatedness among these organisms was drawn using figtree v1.4.3. To analyze the conserved regions and the active site of the protein, Clustal Omega alignment of tyrosine aminotransferase from *Leishmania infantum*,* Leishmania donovani*,* Homo sapiens*,* Trypanosoma cruzi,* and *Rattus norvegicus* was represented through espript 3.0 23. The identical sequences are represented in red, and gaps are shown in dotted line.

### Cloning, expression, and purification of LdTAT

LdTAT of size 1347 bp was amplified from *Leishmania donovani* genomic DNA using gene‐specific forward primer (ATATTA**GGATCC**ATGACGATTGATACGCAG) and reverse primer (GATTTC**AAGCTT**CTACTTCTTGTGGCGCTC). All other chemicals were procured from Sigma‐Aldrich (St.Louis, MO, USA) and Merck KGaA (Darmstadt, Germany) unless specified. The PCR‐amplified product and pET28a(+) (Novagen, Madison, WI, USA) vector were digested using *Bam*HI (NEB) and *Hind*III (NEB) restriction enzymes and ligated together using T4 DNA ligase (NEB) at 4 °C overnight. Transformation of the ligation mixture was conducted on *E. coli* (DH5α) strain. Kanamycin agar plates were employed for the selection of positive clones, which were further confirmed by colony PCR and restriction digestion. The cloned plasmids were then transformed into *E. coli* BL21 strain for higher expression. The transformed *E. coli* BL21 strain was grown till 0.6 OD, and 0.5 mm of IPTG was used for the expression of the protein at 30 °C overnight. The cells were harvested and sonicated in lysis buffer (20 mm Tris/HCl, 500 mm NaCl, and 50 mm imidazole; pH 8.0) for 50 pulses (5 s on/ 10 s off). The sample was further centrifuged, and the supernatant was passed through Ni‐NTA column. The purified protein was then eluted using 500 mm of imidazole. Further dialysis of the protein was carried out against Tris/HCL 10 mm and NaCl 200 mm to remove traces of imidazole. The protein concentration was determined using Bradford's test 24. The purified protein was then transferred onto polyvinylidene fluoride (PVDF) membrane and blocked with 5% skimmed milk solution after transfer. The membrane was then washed with TBST (Tris‐buffered saline, Tween‐20) followed by the addition of anti‐His antibody (1 : 5000) and incubation overnight. The membrane was further incubated with anti‐goat secondary horseradish peroxidase antibody (1 : 10 000), and bands were detected in a Chemidoc by addition of chemiluminescent HRP substrate 25.

### Modeling, docking, and dynamic simulation of LdTAT

The translated sequence of LdTAT was obtained and modeled using Modeller v9.19 26. The template that was chosen for modeling was tyrosine aminotransferase from *Leishmania infantum,* which showed good similarity and identity with LdTAT amino acid sequence on multiple sequence alignment. Over 50 homology models were constructed, and the best models were chosen using GA341 and DOPE scores 27, 28. The top 5 models were then structurally validated using SAVES validation package 29, 30 and RAMPAGE server. The best model was then deposited to Protein Model Database 31, and PMDB ID: PM0081305 was assigned. The structure was then used for further docking and simulation using autodock v4.2 32 and gromacs package v5.1.4 33, respectively. Kollman charge and gasteiger charge were assigned to the LdMPK4 and ligands, respectively, in all experiments. The structure was energy minimized using Amber 99 force field in GROMACS. For Autodock, the grid box was set at 54 × 64 × 64 points around K286 active site cavity. The grid maps were produced for both protein and ligands (PLP and pyridoxal) with electrostatic and desolvation maps. Final docking was performed for 500 LGA runs. For MD simulations, ligand topologies were generated using ACPYPE 34 and they were manually checked for errors. All simulations were solvated by TIP4 water molecules in a dodecahedron box. The system was then neutralized by the addition of 8 Na^+^ ions and energy minimized by steepest gradient integrator. Temperature and pressure equilibration were performed for 5 ns and analyzed by using Modified Berendsen thermostat and Parrinello‐Rahman barostat, respectively. Production MD was then carried out, and trajectory and graphical analysis was conducted using UCSF Chimera 35 and Xmgrace, respectively.

### Kinetic characterization of LdTAT

Tyrosine aminotransferase is a broad aminotransferase that shows activity toward aromatic amino acids and numerous oxo‐acids. The activity assay was performed according to Diamondstone, T.I, 1966 36. Briefly, the enzyme was incubated with the substrate and pyridoxal 5′‐phosphate (PLP) (30 μm) for 1 h at 37 °C, and then, the co‐substrate was added and followed by incubation for 10 min. The reaction was terminated by the addition of 10 N NaOH, and the absorbance was measured at 331 nm after 30 min. All experiments were carried out in triplicated. 50 μg of the enzyme was utilized for all the assays performed. LdTAT was tested for activity toward tyrosine, tryptophan, phenylalanine, sodium pyruvate, α‐ketoglutarate, and 2‐oxo‐4‐methylthiobutanoate (KMB). TAT is a PLP‐dependent enzyme, and PLP is covalently attached to the Ɛ‐amino group of K286 to form an internal aldimine by Schiff‐base interaction. To determine the specificity of the cofactor to the enzyme, both PLP and pyridoxal were assayed for activity using LdTAT and tyrosine–sodium pyruvate were maintained as substrates. PLP structurally differs from pyridoxal by the lack of a phosphate tail. Both PLP and pyridoxal were, therefore, docked with LdTAT, and furthermore, they were simulated with the modeled LdTAT for 100 ns. Hydrogen bond formation between LdTAT and the two molecules was then analyzed.

### Effect of temperature and pH on enzyme activity

To determine the stability and activity of the enzyme at various pH and temperature, LdTAT was subjected to activity assay at pH range of 2–11 and temperature from 20 to 100 °C. All the assays were performed using 4 mm tyrosine and 100 mm of sodium pyruvate. The pH of the assay was varied using different buffers like citrate buffer (pH: 3 and 4), phosphate buffer (pH: 6), HEPES buffer (pH: 7), Tris/HCl (pH: 8), MOPS buffer (pH: 9), and sodium carbonate buffer (pH: 10.6). Effect of temperature was evaluated during the 10‐min incubation period with co‐substrate. The effect of temperature on LdTAT was also studied at the molecular level by simulating the modeled LdTAT enzyme at 27, 40, 50, 60, 70, 80, 90, and 100 °C. RMSD (Root Mean Square Deviation) values for Cα atoms were calculated at different temperatures for a production MD run of 30 ns.

### Folding mechanism using urea denaturation studies

To understand the stability of LdTAT in the presence of chaotropic agents and its folding mechanism, urea‐based denaturation studies were carried out. LdTAT activity was determined at various concentration of urea from 0 to 8 m urea using saturation concentrations of tyrosine and pyruvate as mentioned in the previous section. The relative activity of LdTAT was then calculated accordingly. LdTAT consists of 12 Tyr and 6 Trp residues, and these residues are distributed well throughout the structure. We measured the intrinsic fluorescence of LdTAT to analyze its unfolding mechanism. The fluorescence study was carried using Horiba Jobin‐Yvon Fluorolog‐3 (Model FL 3‐21) equipped with a 1‐cm quartz cell, while the excitation and emission slits were set at 2 nm bandwidth; 0.4 mg·mL^−1^ of LdTAT was used for all experiments that were carried out at 25 ± 1 °C. The excitation wavelength of 295 nm was chosen, and the emission spectrum was measured in the range of 340–550 nm 37. For each sample, measurements were taken in triplicated and the mean was calculated and plotted for analysis. To analyze the different states of folding at the molecular level, the modeled LdTAT was simulated at 8 m urea by adding 3577 molecules of urea and then solvating with TIP4 water molecules. After temperature and pressure equilibration for 5 ns each, production MD was run for 200 ns. The RMSD values of Cα atoms, RMSF (Root Mean Square Fluctuation) of the residues, radius of gyration (Rg), and SASA (Solvent accessible surface area) for Cα atoms were calculated to understand the different folding states that existed in LdTAT.

## Conflict of interest

The authors declare no conflict of interest.

## Author contributions

PS designed and directed the work; SS performed all the experiments; PS and SS contributed to the interpretation of the results. SS wrote the manuscript in consultation with PS.

## Disclosure statement

No potential conflict of interest was reported by the authors.

## Supporting information


**Fig. S1.** Michaelis Menten plot for substrate:co‐substrate pair.
**Fig. S2.** Solvent accessible surface area (SASA) of urea denaturated LdTAT at different unfolding states.

## Data Availability

GenBank id: MK426678; PMDB id: PM0081305.
