# Supplementary material for: Biochemical and structural characterization of tyrosine aminotransferase suggests broad substrate specificity and a two‐state folding mechanism in Leishmania donovani
Source: FEBS Open Bio. 2019 Aug 31;9(10):1769–83. doi: 10.1002/2211-5463.12715 (PMC6768288; doi:10.1002/2211-5463.12715)
Supplement: Supplementary file 1 — Fig. S1. Michaelis Menten plot for substrate:co‐substrate pair. Fig. S2. Solvent accessible surface area (SASA) of urea denaturated LdTAT at different unfolding states. [file FEB4-9-1769-s001.pdf]

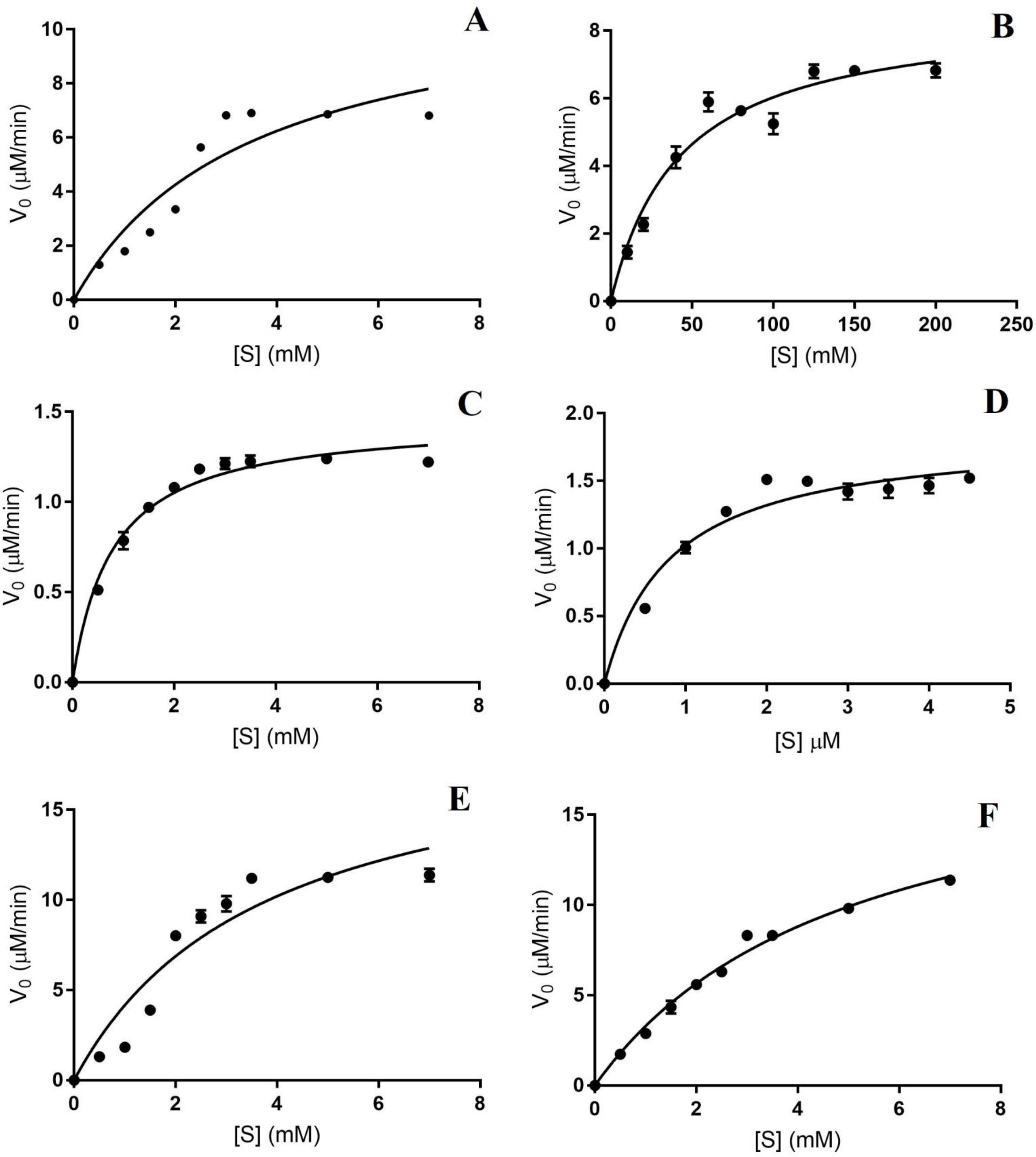

Figure S1: Michaelis Menten plot for substrate:co-substrate pair: (A) SP (100 mM):Tyr (0-7mM) (**B)** Tyr (4 mM):SP (0-200 mM) **(C)** KMB (1.25 μM):Tyr (0-7 mM) **(D)** Tyr (4 mM):KMB (0-4.5 μM) **(E)** SP (100 mM):Phe (0-7 mM) **(F)** SP (100 mM):Trp (0-7 mM). **SP**-Sodium pyruvate; **Tyr-**Tyrosine; **KG-** α-ketoglutarate; **KMB-** α-ketomethiobutyrate; **Phe-**Phenylalanine; **Trp-**Tryptophan.

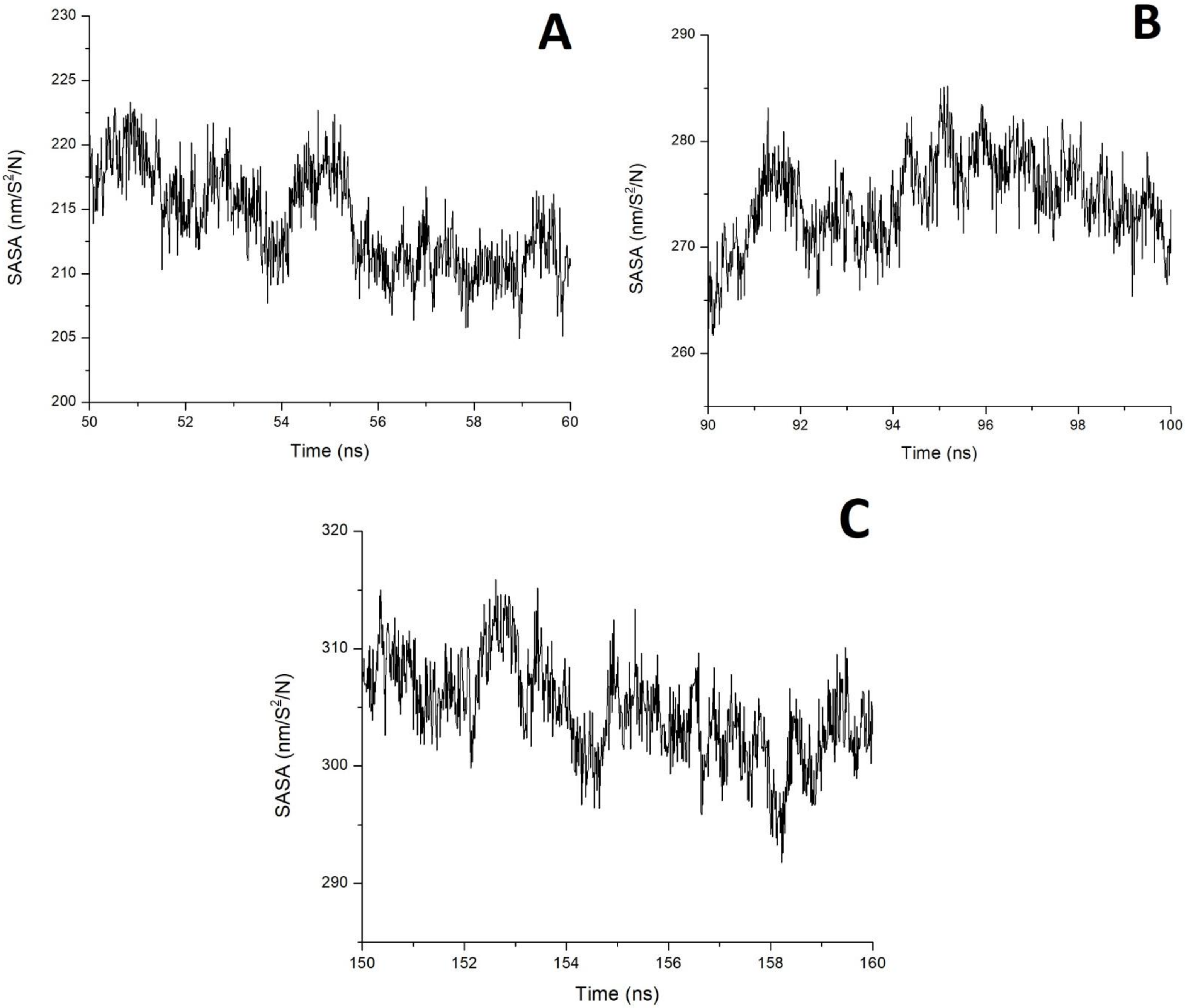

Figure S2: Solvent accessible surface area (SASA) of urea denaturated LdTAT at different unfolding states. (A) SASA analysis at 50 ns. **(B)** SASA analysis at 100 ns **(C)** SASA analysis at 150 ns. The three trajectories extracted at different 10ns intervals displayed increasing solvent accessibility at different stages. This is due to the denaturation and loss of structure in the presence of urea. The difference in the SASA measurements goes in line with the proposed N$\rightarrow$X$\rightarrow$D unfolding mechanism.
